# Development and Use of a Calculator to Measure Pediatric Low-Value Care Delivered in US Children’s Hospitals

**DOI:** 10.1001/jamanetworkopen.2021.35184

**Published:** 2021-12-30

**Authors:** Samantha A. House, Matthew Hall, Shawn L. Ralston, Jennifer R. Marin, Eric R. Coon, Alan R. Schroeder, Heidi Gruhler De Souza, Amber Davidson, Patti Duda, Timmy Ho, Marquita C. Genies, Marcos Mestre, Mario A. Reyes

**Affiliations:** 1 Department of Pediatrics, Geisel School of Medicine, Dartmouth College, Hanover, New Hampshire; 2 Children’s Hospital at Dartmouth-Hitchcock Medical Center, Lebanon, New Hampshire; 3 Children’s Hospital Association, Lenexa, Kansas; 4 Department of Pediatrics, University of Washington, Seattle; 5 UPMC Children’s Hospital of Pittsburgh, Pittsburgh, Pennsylvania; 6 Department of Pediatrics, University of Utah, Salt Lake City; 7 Department of Pediatrics, Stanford University, Stanford, California; 8 Division of Newborn Medicine, Department of Pediatrics, Boston Children’s Hospital, Boston, Massachusetts; 9 Department of Neonatology, Beth Israel Deaconess Medical Center, Boston, Massachusetts; 10 Department of Pediatrics, Harvard Medical School, Boston, Massachusetts; 11 Department of Pediatrics, Johns Hopkins Medical School, Baltimore, Maryland; 12 Department of Pediatrics, Division of Hospital Medicine, Nicklaus Children’s Hospital, Miami, Florida

## Abstract

**Importance:**

The scope of low-value care in children’s hospitals is poorly understood.

**Objective:**

To develop and apply a calculator of hospital-based pediatric low-value care to estimate prevalence and cost of low-value services.

**Design, Setting, and Participants:**

This cross-sectional study developed and applied a calculator of hospital-based pediatric low-value care to estimate the prevalence and cost of low-value services among 1 011 950 encounters reported in 49 US children’s hospitals contributing to the Pediatric Health Information System (PHIS) database. To develop the calculator, a multidisciplinary stakeholder group searched existing pediatric low-value care measures and used an iterative process to identify and operationalize relevant hospital-based measures in the PHIS database. Children with an eligible encounter in 2019 were included in the calculator-applied analysis. Two cohorts were analyzed: an emergency department cohort (with encounters resulting in emergency department discharge) and a hospitalized cohort.

**Exposures:**

Eligible condition-specific hospital encounters.

**Main Outcomes and Measures:**

The proportion and volume of encounters in which low-value services were delivered and their associated standardized costs. Measures were ranked by those outcomes.

**Results:**

There were 1 011 950 encounters eligible for 1 or more of 30 calculator-included measures in 2019; encounters were incurred by 816 098 unique patients with a median age of 3 years (IQR, 1-8 years). In the emergency department cohort, low-value services delivered in the greatest percentage of encounters were Group A streptococcal testing among children younger than 3 years with pharyngitis (3679 of 9785 [37.6%]), computed tomography scan for minor head injury (7541 of 42 602 [17.7%]), and bronchodilators for treatment of bronchiolitis (8899 of 55 616 [16.0%]). In the hospitalized cohort, low-value care was most prevalent for broad-spectrum antibiotics in the treatment of community-acquired pneumonia (3406 of 5658 [60.2%]), acid suppression therapy for infants with esophageal reflux (3814 of 7507 of [50.8%]), and blood cultures for uncomplicated community-acquired pneumonia (2277 of 5823 [39.1%]). Measured low-value services generated nearly $17 million in total standardized cost. The costliest services in the emergency department cohort were computed tomography scan for abdominal pain (approximately $1.8 million) and minor head injury (approximately $1.5 million) and chest radiography for asthma (approximately $1.1 million). The costliest services in the hospitalized cohort were receipt of 2 or more concurrent antipsychotics (approximately $2.4 million), and chest radiography for bronchiolitis ($801 680) and asthma ($625 866).

**Conclusions and Relevance:**

This cross-sectional analysis found that low-value care for some pediatric services was prevalent and costly. Measuring receipt of low-value services across conditions informs prioritization of deimplementation efforts. Continued use of this calculator may establish trends in low-value care delivery.

## Key Points

**Question:**

What are the prevalence and cost associated with a set of low-value services across US children's hospitals?

**Findings:**

This cross-sectional study used an evidence-based low-value care calculator to estimate prevalence and cost of low-value services among 1 011 950 encounters across 49 children’s hospitals contributing to the Pediatric Health Information System database. The prevalence of low-value care ranged from less than 1% to 60% across measures, with nearly $17 million in standardized cost attributable to 30 measured low-value services.

**Meaning:**

This study found that low-value care was costly, but prevalence varied widely across measured services; use of this calculator may aid in prioritization of deimplementation initiatives.

## Teaser

This cross-sectional study uses a calculator of hospital-based pediatric low-value care to estimate the proportion and volume of low-value services and their associated standardized costs delivered in US children’s hospitals.

## Introduction

Low-value care, or delivery of health services offering limited benefit as compared with harm, is an important domain of health care waste.^1,2,3^ Consequences associated with such care range from physical effects, including adverse medication effects and procedural complications, to psychosocial and financial effects of incidental findings, false diagnoses, and downstream health care utilization. The prevalence and impact of low-value care remain poorly understood in pediatrics; measurement has proven challenging owing to the number and diversity of low-value practices, fragmented data sources, and a dearth of quality measures focused on low-value service delivery.^4,5,6^

Administrative databases containing billing information for a large number of encounters and offering accessible data for longitudinal measurement have emerged as sources for quantifying low-value care.^7,8,9^ One proprietary tool measuring nearly 50 low-value services primarily delivered to adults has been applied to state- and payer-level data sets, identifying common and costly services and describing temporal low-value care trends.^7,9,10^ Studies using administrative data have established low-value care as an important pediatric problem,^11,12,13,14,15^ but most studies describe care at a single time point or for a limited set of measures. With child health spending estimated to be equivalent to half the US defense budget at more than $300 billion^16^ and increasing recognition of harms associated with low-value care, understanding the extent of this problem in pediatrics is imperative. Tools leveraging large data sources for longitudinal measurement of low-value care and benchmark setting may prove valuable in scoping the issue.

As strategies to measure pediatric low-value care evolve, hospital-based care warrants particular attention. This care is increasingly costly,^17^ and literature on overuse of nonrecommended hospital-based pediatric services is robust, suggesting improvement opportunities.^14,15,18,19,20,21^ Given this context, our specific aim was to develop a calculator to measure low-value care within US children’s hospitals. In this report, we describe the development of this calculator and apply the calculator to estimate prevalence and cost associated with low-value services in US children’s hospitals during 2019.

## Methods

### Development of the Low-Value Care Calculator

#### Overview

Following the principles of the National Quality Forum for quality measure set development,^22^ we convened a multidisciplinary stakeholder group of 9 subject matter experts (SMEs) consisting of pediatricians practicing hospital medicine (S.A.H., S.L.R., E.R.C., A.R.S., M.C.G., M.M., and M.A.R.), emergency medicine (J.R.M.), critical care (A.R.S.), and neonatology (T.H.) to develop a low-value care calculator. All SMEs had research experience with health care value and quality measurement. We identified and operationalized evidence-based low-value care measures and recommendations within the Pediatric Health Information System (PHIS; Children’s Hospital Association [CHA], Lenexa, Kansas) database. We selected inpatient, emergency department (ED), and neonatal intensive care unit (NICU) settings as our areas of focus owing to availability of measures relevant to these settings. The calculator development process is outlined in the Figure.^11,23^ This study followed the Strengthening the Reporting of Observational Studies in Epidemiology (STROBE) reporting guideline^24^ and was deemed not human subjects research by the Dartmouth College Institutional Review Board.

**Figure.**
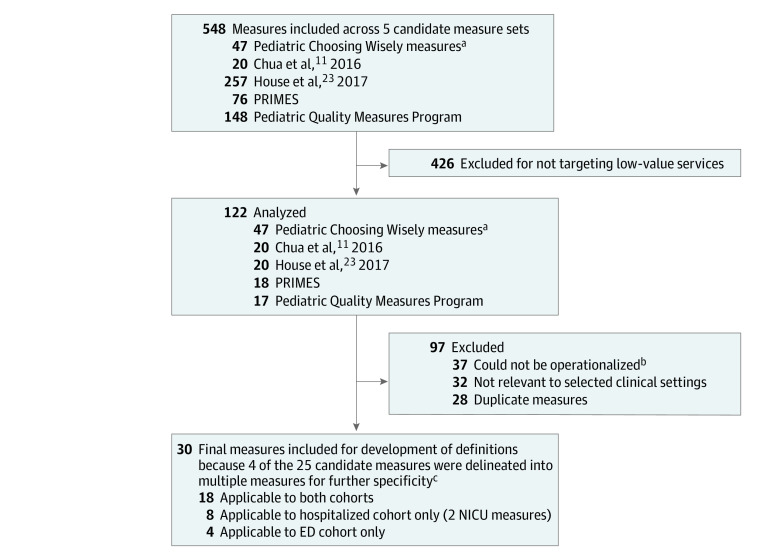
Measure Selection Process ED indicates emergency department; NICU, neonatal intensive care unit; and PRIMES, Pediatric Respiratory Illness Measurement System. ^a^Pediatric Choosing Wisely measures include all recommendations published in the Choosing Wisely campaign at the time of our measure search that were potentially applicable to pediatric populations. ^b^Available administrative data considered not adequate to define measures with fidelity to original measure intent. ^c^Single head-imaging measures for febrile seizure and headache as 2 unique measures (computed tomography and magnetic resonance imaging); single laboratory measure for febrile seizure as 2 unique measures (complete blood count and electrolytes); and single measure for peripherally inserted central catheter placement for complicated infections as 3 measures (bone and joint infections, complicated pneumonia, and ruptured appendicitis).

#### Measure Selection

We first identified published pediatric quality measures or recommendations targeting reduction of nonevidence-based services (ie, low-value care measures). Relevant measure sources were identified using the Measure Use Tool from CHA,^25^ which is a repository of pediatric quality measures, and through peer-reviewed literature describing or categorizing pediatric quality measures. We identified 5 candidate sources including low-value care measures (Figure).^11,23,26,27,28^ Two sources contained only low-value care measures^11,26^; 2 other sources categorized measures by type, explicitly identifying low-value care measures.^23,28,29^ The Pediatric Quality Measures Program measures^27^ were not categorized; 3 authors (S.A.H., S.L.R., and M.A.R.) categorized these measures to identify those targeting low-value service delivery.

After duplicate measures were excluded, a set of unique low-value care measures was distributed to all SMEs. The SMEs were first asked to determine whether each measure was relevant in at least 1 target clinical setting. Measures for which there was universal agreement on setting applicability continued to the next round of review (if deemed applicable) or were removed (not applicable). All other measures were iteratively discussed until consensus was reached. This method was then repeated, with SMEs determining whether individual measures could be operationalized within PHIS. Measures were excluded if SMEs felt that the clinical information needed to determine whether a service was low value was more nuanced than that provided in PHIS. Final candidate measures were then reviewed with members of the PHIS analytic team (M.H., H.G.D.S., A.D., and P.D.) to ensure feasibility of operationalization within the database.

#### Measure Construction

Our SME group determined an approach to measure construction a priori. For measures with clear specifications, we matched original inclusion and exclusion criteria as closely as possible. For measures without clear specifications or measures appearing in multiple measure sets with conflicting specifications, we constructed definitions that were as narrow, or specific, as possible. Prior literature shows that estimates of low-value care vary with approach to measure definition.^11^ Narrow measure definitions with multiple restrictions prioritize specificity, capturing care that is likely to be low-value but potentially underestimating low-value service delivery; broader measures with minimal restrictions prioritize sensitivity while potentially misidentifying some appropriate use as low value.^11^ The narrow measures we used were intended to capture consensus-defined low-value care and to minimize misclassification of appropriate care, acknowledging possible underestimation of low-value care for some measures.

For all measures, we excluded patients older than 18 years and patients with *International Statistical Classification of Diseases, Tenth Revision, Clinical Modification* (*ICD-10-CM*) codes documenting a complex chronic condition^30^ or neurologic impairment^31^ within the year prior to the included encounter. For hospitalized patients, we additionally excluded encounters with an All Patient Refined Diagnosis Related Group (3M) extreme severity of illness and patients admitted to an intensive care unit at any point during hospitalization (with the exception of NICU-specific measures). These exclusions were determined a priori given that the primary literature sources supporting included measures often exclude these populations.

Measure definitions are shown in the eTable in the Supplement. Inclusion and exclusion criteria were derived from *ICD-10-CM* and *Current Procedural Terminology* codes. To achieve narrow definitions, we excluded encounters with diagnostic codes that SMEs felt may justify service delivery. Clinical services were defined by Clinical Transaction Classification codes specific to PHIS.

### Data Source and Study Design

After calculator development, we conducted a cross-sectional, observational cohort study using the PHIS database. This database contains deidentified administrative data detailing demographic characteristics, diagnostics, procedures, and daily billing information from 49 tertiary referral care children’s hospitals, accounting for approximately 20% of all annual pediatric hospitalizations and approximately 12% of all ED visits in the US. Data quality is ensured through a joint effort between CHA and participating hospitals.

Results were analyzed for 2 cohorts: (1) the ED cohort, including encounters resulting in discharge from the ED, and (2) the hospitalized cohort, including encounters for patients admitted to a medical department (inpatient or observation status) or to the NICU. In the hospitalized cohort, care delivered during the inpatient encounter was not separable from that delivered in the associated ED encounter within the same center; as such, results for the hospitalized cohort reflect care delivered in both settings, if applicable.

Data were analyzed from January 1 to December 31, 2019, and hospitals were included only if they consistently contributed data during this period. Encounters were eligible if they met inclusion criteria for at least 1 included measure and no exclusions were identified.

### Calculator Outcomes

We used the low-value care calculator to assess 3 outcomes for each measure: (1) percentage of eligible encounters in which a low-value service was delivered, (2) number of encounters in which a low-value service was delivered, and (3) standardized unit cost associated with low-value care. Standardized unit costs were previously developed by the CHA as a measure for comparison of resource utilization across hospitals in the setting of interhospital variation in cost definitions and are determined by calculating the median cost for services across PHIS hospitals; a full description is published elsewhere.^32^

### Statistical Analysis

To inform deimplementation efforts, we ranked measures in each setting by these 3 outcomes. As a subanalysis, we grouped measures into 4 categories (medications, imaging, labs, and procedures) and calculated standardized cost associated with low-value care for each category. We also calculated category-specific standardized cost for all eligible encounters (ie, a sum of cost for medications provided in all eligible encounters), allowing determination of the percentage of total standardized cost within a category that was attributable to low-value care. Statistical analyses were performed with SAS, version 9.4 (SAS Institute Inc).

## Results

The final low-value care calculator included 30 measures. Of these measures, 22 were applicable to the ED cohort; 774 584 encounters by 621 633 unique patients were eligible for these measures from 47 hospitals. There were 26 measures applicable to the hospitalized cohort (including 2 NICU measures), for which there were 237 366 eligible encounters by 194 465 patients from 49 hospitals. The median age of patients with included encounters was 3 years (IQR, 1-8 years).

### ED Cohort

Table 1 describes low-value care delivery for the ED cohort. Measures with the greatest percentage of low-value care delivery among eligible encounters were testing for group A streptococcus among children younger than 3 years with pharyngitis (3679 of 9785 [37.6%]), computed tomography (CT) scan for minor head injury (7541 of 42 602 [17.7%]), and bronchodilator treatment of bronchiolitis (8899 of 55 616 [16.0%]).

**Table 1.** Low-Value Care Prevalence and Associated Standardized Cost, Emergency Department Cohort

Condition	Measure		Eligible encounters, No.	Encounters with low-value care delivered				Standardized cost associated with low-value service, $	Rank by cost of low-value care
	Population	Low-value service		%	Rank by %	No.	Rank by No.		
Pharyngitis	<3 y of Age treated in ED for pharyngitis	Testing for GAS pharyngitis unless other risk factors present	9785	37.6	1	3679	7	80 151	14
Head injury	Treated in ED for minor head injuries	CT imaging of the head	42 602	17.7	2	7541	4	1 517 548	2
Bronchiolitis	Diagnosed as having bronchiolitis	Bronchodilator treatment	55 616	16.0	3	8899	2	473 918	7
Bronchiolitis	Diagnosed as having bronchiolitis	Chest radiography	55 616	15.6	4	8676	3	926 958	6
Asthma	Diagnosed as having asthma	Chest radiography	71 239	15.4	5	10 971	1	1 092 793	3
Seizure	Diagnosed as having incident generalized afebrile atraumatic seizure	CT imaging of the head	8646	13.8	6	1193	13	230 339	8
Pneumonia	Diagnosed as having uncomplicated CAP	Antibiotic therapy broader than ampicillin	25 197	13.6	7	3427	8	180 167	9
Headache	Treated in ED for acute atraumatic primary headache	CT imaging of the head	39 802	12.2	8	4856	5	1 042 794	4
Pneumonia	Diagnosed as having uncomplicated CAP	Bacterial blood culture	25 170	7.3	9	1837	10	151 261	11
Febrile seizure	Diagnosed as having simple febrile seizure	CBC testing	10 388	7.1	10	738	15	54 978	15
Pneumonia	Diagnosed as having uncomplicated CAP	C-reactive protein and erythrocyte sedimentation rate tests	25 255	6.4	11	1616	12	115 935	12
Febrile seizure	Diagnosed as having simple febrile seizure	Electrolyte testing	10 388	5.9	12	613	17	51 474	16
Headache	Treated in ED for acute atraumatic primary headache	MRI of the head	39 802	4.5	13	1791	11	1 038 481	5
Bronchiolitis	Diagnosed as having bronchiolitis	Treatment with corticosteroids	55 616	4.3	14	2391	9	151 848	10
Abdominal pain	With abdominal pain	CT imaging of abdomen unless other indications present	122 084	3.8	15	4639	6	1 767 120	1
Gastroesophageal reflux	Age <1 y with gastroesophageal reflux	Acid suppression therapy	11 619	2.3	16	267	18	2167	22
Bronchiolitis	Diagnosed as having bronchiolitis	Bacterial blood culture	52 444	1.4	17	734	16	80 745	13
Febrile seizure	Diagnosed as having simple febrile seizure	CT imaging of the head	10 872	1.0	18	109	21	24 138	18
Bronchiolitis	Diagnosed as having bronchiolitis	Antibiotic medications unless also diagnosed as having possible bacterial infection	46 639	0.3	19	140	19	13 076	19
Viral respiratory infection	Diagnosed as having viral upper respiratory tract infection	Antibiotic medications unless also diagnosed as having possible bacterial infection	377 064	0.3	20	1131	14	41 229	17
Asthma	Diagnosed as having asthma	Antibiotic medications unless also diagnosed as having possible bacterial infection	69 040	0.2	21	138	20	8518	20
Febrile seizure	Diagnosed as having simple febrile seizure	MRI of the head	10 872	0.1	22	11	22	2928	21

Abbreviations: CAP, community-acquired pneumonia; CBC, complete blood count; CT, computed tomography; GAS, group A streptococcus; ED, emergency department; MRI, magnetic resonance imaging.

Measures for which low-value care was associated with the greatest number of encounters were chest radiography for asthma (n = 10 971), followed by bronchodilators for treatment of bronchiolitis (n = 8899), and chest radiography for bronchiolitis (n = 8676). The measures associated with the greatest cost were CT scan for abdominal pain (approximately $1.8 million), CT scan for minor head injury (approximately $1.5 million), and chest radiography for asthma (approximately $1.1 million). Magnetic resonance imaging for febrile seizure (n = 11; $2928) and antibiotics for treatment of asthma (n = 138; $8518) were measures for which low-value care delivery was infrequent and associated costs were low.

### Hospitalized Cohort

Table 2 describes low-value care delivery for the hospitalized cohort. Measures with the greatest percentage of low-value care delivery among eligible encounters were antibiotics broader than ampicillin for treatment of community-acquired pneumonia (CAP; 3406 of 5658 [60.2%]), acid suppression therapy for infants younger than 1 year with esophageal reflux (3814 of 7507 [50.8%]), and blood cultures for uncomplicated CAP (2277 of 5823 [39.1%]).

**Table 2.** Low-Value Care Prevalence and Associated Standardized Cost, Hospitalized Cohort

Condition	Measure		Eligible encounters, No.	Encounters with low-value care delivered				Standardized cost associated with low-value service, $	Rank by cost of low-value care
	Population	Low-value service		%	Rank by %	No.	Rank by No.		
**Inpatient or observation**									
Pneumonia	Diagnosed as having uncomplicated CAP	Antibiotic therapy broader than ampicillin	5658	60.2	1	3406	5	426 279	5
Gastroesophageal reflux	Aged <1 y with gastroesophageal reflux	Acid suppression therapy	7507	50.8	2	3814	4	153 240	13
Pneumonia	Diagnosed as having uncomplicated CAP	Bacterial blood culture	5823	39.1	3	2277	8	316 226	7
Febrile seizure	Diagnosed as having simple febrile seizure	CBC testing	574	35.4	4	203	19	27 549	20
Asthma	Diagnosed as having asthma	Chest radiography	19 145	32.4	5	6203	3	625 866	3
Bronchiolitis	Diagnosed as having bronchiolitis	Treatment with bronchodilators	22 179	31.4	6	6964	1	548 714	4
Febrile seizure	Diagnosed as having simple febrile seizure	Electrolyte testing	574	31.2	7	179	20	28 296	19
Pneumonia	Diagnosed as having uncomplicated CAP	C-reactive protein and erythrocyte sedimentation rate tests	5817	30.4	8	1768	10	252 323	9
Bronchiolitis	Diagnosed as having bronchiolitis	Chest radiography	22 179	28.2	9	6254	2	801 680	2
Behavioral health	Receiving antipsychotic medications	≥2 Antipsychotic medications concurrently	15 745	20.8	10	3275	6	2 384 334	1
Abdominal pain	With abdominal pain	CT scan of the abdomen unless other indications are present	6807	12.4	11	844	12	334 334	6
Pneumonia	Diagnosed as having complicated pneumonia	Should not have PICC or CVL placement for extended intravenous antibiotic therapy; oral conversion to antibiotics preferred to PICC or CVL	2214	11.8	12	261	18	204 894	11
Bronchiolitis	Diagnosed as having bronchiolitis	Treatment with corticosteroids	22 179	10.4	13	2307	7	288 709	9
Bronchiolitis	Diagnosed as having bronchiolitis	Bacterial blood cultures	19 101	9.3	14	1776	9	239 824	10
Seizure	Diagnosed as having incident generalized afebrile atraumatic seizure	CT imaging of the head	3977	9.1	15	362	17	71 553	15
Febrile seizure	Diagnosed as having simple febrile seizure	CT imaging of the head	654	6.7	16	44	22	11 839	22
Viral respiratory infection	Diagnosed as having viral upper respiratory infection	Antibiotic medications unless also diagnosed as having possible bacterial infection	27 825	6.0	17	1670	11	163 357	12
Febrile seizure	Diagnosed as having simple febrile seizure	MRI of the head	654	5.8	18	38	23	23 013	21
Bone and joint infections	Diagnosed as having bone and joint infections	PICC or CVL placement for extended intravenous antibiotic therapy; oral conversion to antibiotics preferred to PICC or CVL	7531	5.3	19	399	16	303 697	8
Bronchiolitis	Diagnosed as having bronchiolitis	Antibiotic medications unless also diagnosed as having possible bacterial infection	15 346	4.3	20	660	13	94 034	14
Appendicitis	Diagnosed as having ruptured appendicitis	PICC or CVL placement for extended intravenous antibiotic therapy; oral conversion to antibiotics preferred to PICC or CVL	837	3.7	21	31	24	29 410	18
Asthma	Diagnosed as having asthma	Antibiotic medications unless also diagnosed as having possible bacterial infection	18 417	2.2	22	405	14	44 374	17
Asthma	Admitted to the hospital with acute exacerbation of asthma	Ipratropium bromide after 24 h of hospitalization	19 145	2.1	23	402	15	53 566	16
Behavioral health	<5 y of Age	Antipsychotic medication	109 538	0.1	24	110	21	9787	23
**Neonatal intensive care**									
Neonatal intensive care	Infants in the NICU	Antireflux medication for treatment of symptomatic GERD or of apnea and desaturation	230	13.9	1	32	2	2186	2
Neonatal intensive care	Infants in the NICU	Vancomycin or carbapenems unless there is known risk for resistant pathogens	25 543	2.5	2	639	1	82 408	1

Abbreviations: CAP, community-acquired pneumonia; CBC, complete blood count; CT, computed tomography; CVL, central venous line; GERD, gastroesophageal reflux disease; MRI, magnetic resonance imaging; NICU, neonatal intensive care unit; PICC, peripherally inserted central line.

Measures for which low-value care was associated with the greatest number of encounters was bronchodilator treatment of bronchiolitis (n = 6964) and chest radiography for bronchiolitis (n = 6254) and asthma (n = 6203). The costliest measures were receipt of 2 or more concurrent antipsychotics (approximately $2.4 million), chest radiography for bronchiolitis ($801 680) and chest radiography for asthma ($625 866).

Measures showing the lowest proportion of low-value care delivery included antipsychotics for children younger than 5 years (110 of 109 538 eligible encounters [0.1%]), ipratropium bromide after 24 hours of hospitalization for treatment of asthma (402 of 19 145 [2.1%]), and antibiotics for treatment of asthma (405 of 18 417 [2.2%]). The NICU measures were ranked separately (Table 2).

### Cost by Condition and by Category

Across all conditions, measured low-value care delivery generated nearly $17 million in standardized cost; 55% of this cost was generated by low-value services in the ED cohort. Bronchiolitis measures generated the greatest standardized cost at more than $3.6 million, followed by behavioral health measures at nearly $2.4 million. Low-value care for pneumonia, abdominal pain, and asthma generated substantial cost in both cohorts (Table 3). The median standardized cost of low-value care per hospital was $306 018 (IQR, $157 397-$481 965).

**Table 3.** Cost Associated With Low-Value Care by Condition and Clinical Setting

Condition	Measures, No.^a^	Eligible encounters, No.^b^	Total standardized unit cost, $		Total standardized unit cost, $
			Emergency department cohort	Hospitalized cohort	
Bronchiolitis	5	77 795	1 646 545	1 972 961	3 619 506
Behavioral health	2	109 538		2 394 121	2 394 121
Abdominal pain	1	128 891	1 767 120	334 334	2 101 454
Headache	2	39 802	2 081 275		2 081 275
Asthma	3	90 384	1 101 311	723 806	1 825 117
Pneumonia	4	31 072	447 363	1 199 722	1 647 085
Head Injury	1	42 602	1 517 548		1 517 548
Bone and joint infection	1	7531		303 697	303 697
Seizure	1	12 623	230 339	71 553	301 892
Febrile Seizure	4	11 526	133 518	90 697	224 215
Viral respiratory infection	1	404 889	41 229	163 357	204 586
Gastroesophageal reflux	1	19 126	2167	153 240	155 407
Pharyngitis	1	9785	80 151		80 151
Appendicitis	1	837		29 410	29 410
Neonatal intensive care measures	2	25 543		84 594	84 594
Total			9 048 566	7 521 492	16 570 058

^a^ Includes number of unique measures applicable to either emergency department or hospitalized cohorts.

^b^ Eligible encounters represent the total number of encounters eligible for any measure within each condition.

Table 4 gives the standardized cost associated with each category of low-value care. The 9 imaging measures accounted for the largest standardized cost overall (>$9.5 million) and the greatest proportional cost by category, accounting for 27.3% of all standardized imaging costs among eligible encounters.

**Table 4.** Standardized Cost of Low-Value Care by Category

Category	Measures, No.	Standardized cost, $		% of Total cost attributed to low-value care
		Low-value care	Total category	
Medication	12	5 121 911	148 845 830	3.4
Imaging	9	9 511 384	34 903 836	27.3
Laboratory tests	6	1 398 762	18 693 290	7.5
Invasive procedures	3	538 001	12 035 580	4.5

## Discussion

The development and application of a calculator incorporating 30 pediatric, hospital-based, low-value care measures revealed nearly $17 million in standardized costs attributable to these practices in 2019. A wide range of performance was observed across measures, with group A streptococcus testing for young children and broad-spectrum antibiotic use for treatment of CAP being delivered in the highest proportion of encounters in the ED and hospitalized cohorts, respectively. Our results support prior assertions that low-value pediatric care warrants focused measurement and improvement efforts.^16^

Our work was informed by prior efforts to describe pediatric low-value care. Chua et al^11^ developed 20 claims-based measures of pediatric low-value care that have been applied to multiple data sources.^12,13^ An analysis of care delivered in 2014 found that at least 10% of commercially insured children received 1 or more of these services, accounting for $27 million in spending; 34% of this total was paid out of pocket.^11^ Modestly higher rates of low-value service delivery were identified among publicly insured and military-insured children.^12,33^ Reyes et al,^14,15^ using the PHIS database, created a report card to measure performance on the original measures included in the pediatric Choosing Wisely Campaign by the Society of Hospital Medicine among hospitalized patients and found that low-value care delivery ranged from 12% to 49% across these measures. Our work incorporates a broad set of hospital-based, pediatric, low-value care measures into a tool capable of sustaining these measurement efforts. The low-value care identified in our study highlights the persistent potential for value improvement in pediatrics through deimplementation of nonevidence-based practices.

Bronchiolitis, CAP, and asthma measures had a relatively high prevalence of low-value care among both cohorts. These are among the most common and costly conditions treated in the pediatric hospital setting^34^ and are popular targets for quality improvement initiatives, yet low-value care persists. Comparisons between our data and those previously published reveal important trends. For example, in the hospitalized cohort, use of broad-spectrum antibiotic therapy for CAP was only slightly lower than that observed in PHIS in 2012,^19^ supporting a need for innovation on this measure. This need is reinforced by the inclusion of this measure in the 2021 Pediatric Hospital Medicine Choosing Wisely recommendations.^35^ Blood culture rates for CAP were even higher than some prior PHIS estimates for the hospitalized cohort.^36^ On the contrary, broad-spectrum antibiotic and blood culture use in the ED were considerably lower than rates previously described in the ED setting using varying data sources.^37,38,39,40^ These results highlight the importance of assessing the trajectory of low-value care over time; our calculator can facilitate the longitudinal measurement needed to establish such trends.

Our investigation also identified low-value care for conditions that have historically not been prioritized for deimplementation. In the inpatient cohort, acid suppression was used in more than one-half of encounters by children with gastroesophageal reflux. This rate is similar to that observed by Reyes et al^14,15^ in 2017. Despite a clinical practice guideline recommending against this treatment,^41^ published quality improvement initiatives targeting this service are limited; inpatient clinicians may be in a unique position to effect change in this practice. Concurrent antipsychotic administration occurred in 21% of eligible encounters. Although multiple psychotropic medications may be deemed necessary to maintain patient and staff safety in some clinical circumstances, evidence for the effectiveness of this practice has not been established, and the potential for harm related to adverse effects and drug-drug interactions is high.^42,43^ Efforts should be made to explore whether additional hospital-based behavioral health resources may decrease this practice.

In the ED, group A streptococcus testing was performed in more than one-third of patients younger than 3 years with pharyngitis. With low rates of pathogenic streptococcal pharyngitis and very low risk of complications, such as acute rheumatic fever, in this population,^44,45^ this practice places children at risk for unnecessary antibiotics and associated adverse effects.

As efforts increase to alleviate measurement burden in health care,^46^ data identifying measures that might be deprioritized are also useful. In the ED cohort, head imaging for febrile seizures and blood cultures for bronchiolitis were observed relatively infrequently in eligible encounters. In the hospital cohort, antipsychotic administration to children younger than 5 years and ipratropium delivery after 24 hours of hospitalization were also infrequent.

This single-year analysis represents an initial step; several future steps may enhance understanding of low-value care patterns in US children’s hospitals. Continued application of this tool will aid in establishing and monitoring temporal low-value care trends and identifying services in need of ongoing deimplementation efforts. Hospital-specific reports have been distributed to PHIS-participating centers to facilitate benchmarking and local quality improvement work. Finally, further analyses will characterize variation in low-value care by hospital and aim to identify facilitators and barriers to value improvement.

### Limitations

Our work has important limitations. Our measure definitions rely on diagnostic codes representing the discharge diagnosis for a particular encounter. These codes are influenced by services provided during the encounter and their findings. As a result, it is possible that some inappropriately prescribed services influenced discharge diagnosis codes such that low-value care was underestimated. For example, our narrow measure definitions would not identify scenarios in which inappropriate chest radiography in the setting of bronchiolitis led to overdiagnosis and overtreatment of pneumonia, as pneumonia is an exclusionary diagnosis for this measure. Conversely, our approach may have overestimated low-value care when appropriately prescribed services return normal results. For example, a significant mechanism of injury or subtle behavioral change in a child with head trauma may warrant a CT scan, but a normal CT scan may result in a diagnosis of minor head injury and thus be deemed of low-value. It is our hope that hospitals will use individualized data in the context of peer hospitals to better understand their practice patterns and track improvements over time. Further efforts to validate included measures with robust clinical data will also strengthen conclusions that can be drawn from calculator use.

In addition, our data reflect only practices associated with published low-value care measures in specific clinical settings; as such, they should not be viewed as a comprehensive picture of all hospital-based low-value care. Our analysis includes only data from US children’s hospitals participating in PHIS, and our findings may not be generalizable to other settings. With a majority of pediatric hospital-based care being delivered outside of these centers, our results reflect a minority of pediatric low-value care delivery. Efforts to expand low-value care measurement beyond the children’s hospital setting are critical to gaining a more robust understanding of how such care may impact children. Finally, we have not assessed harms associated with low-value care beyond direct financial cost; further exploration of outcomes, including related downstream health care utilization, is needed.

## Conclusions

We identified nearly $17 million in cost associated with low-value services delivered in US children’s hospitals during a single year. Our analysis identified some low-value services that are frequent and costly and other low-value services with lesser associated impact, offering data for prioritization of deimplementation efforts.

## Notes

Supplement. **eTable.** Measure Definitions and Sources

## References

[1] Shrank WH, Rogstad TL, Parekh N. Waste in the US health care system: estimated costs and potential for savings. JAMA. 2019;322(15):1501-1509. doi:10.1001/jama.2019.13978 31589283

[2] Badgery-Parker T, Pearson SA, Dunn S, Elshaug AG. Measuring hospital-acquired complications associated with low-value care. JAMA Intern Med. 2019;179(4):499-505. doi:10.1001/jamainternmed.2018.7464 30801628 PMC6450303

[3] Ganguli I, Simpkin AL, Lupo C, . Cascades of care after incidental findings in a US national survey of physicians. JAMA Netw Open. 2019;2(10):e1913325. doi:10.1001/jamanetworkopen.2019.13325 31617925 PMC6806665

[4] Marcotte LM, Schuttner L, Liao JM. Measuring low-value care: learning from the US experience measuring quality. BMJ Qual Saf. 2020;29(2):154-156. doi:10.1136/bmjqs-2019-010191 31649163

[5] Miller G, Rhyan C, Beaudin-Seiler B, Hughes-Cromwick P. A framework for measuring low-value care. Value Health. 2018;21(4):375-379. doi:10.1016/j.jval.2017.10.017 29680091

[6] Newton EH, Zazzera EA, Van Moorsel G, Sirovich BE. Undermeasuring overuse—an examination of national clinical performance measures. JAMA Intern Med. 2015;175(10):1709-1711. doi:10.1001/jamainternmed.2015.4025 26258406

[7] Washington Health Alliance. First, do no harm: calculating health care waste in Washington State: multi-year and medical group results. October 2019. Accessed July 16, 2020. https://www.wacommunitycheckup.org/media/47217/first-do-no-harm-oct-2019.pdf

[8] Mafi JN, Russell K, Bortz BA, Dachary M, Hazel WA Jr, Fendrick AM. Low-cost, high-volume health services contribute the most to unnecessary health spending. Health Aff (Millwood). 2017;36(10):1701-1704. doi:10.1377/hlthaff.2017.0385 28971913 PMC6727655

[9] Mafi JN, Reid RO, Baseman LH, . Trends in low-value health service use and spending in the US Medicare fee-for-service program, 2014-2018. JAMA Netw Open. 2021;4(2):e2037328. doi:10.1001/jamanetworkopen.2020.37328 33591365 PMC7887655

[10] Milliman. MedInsight: health waste calculator. Accessed January 18, 2018. https://www.medinsight.milliman.com/-/media/medinsight/pdfs/medinsight-health-waste-calculator.ashx

[11] Chua KP, Schwartz AL, Volerman A, Conti RM, Huang ES. Use of low-value pediatric services among the commercially insured. Pediatrics. 2016;138(6):e20161809. doi:10.1542/peds.2016-1809 27940698 PMC5127068

[12] Chua KP, Schwartz AL, Volerman A, Conti RM, Huang ES. Differences in the receipt of low-value services between publicly and privately insured children. Pediatrics. 2020;145(2):e20192325. doi:10.1542/peds.2019-2325 31911477 PMC6993279

[13] Koehlmoos TP, Madsen CK, Banaag A, Haider AH, Schoenfeld AJ, Weissman JS. Assessing low-value health care services in the military health system. Health Aff (Millwood). 2019;38(8):1351-1357. doi:10.1377/hlthaff.2019.00252 31381388

[14] Reyes M, Paulus E, Hronek C, . Choosing Wisely Campaign: report card and achievable benchmarks of care for children’s hospitals. Hosp Pediatr. 2017;7(11):633-641. doi:10.1542/hpeds.2017-0029 29066468

[15] Reyes MA, Etinger V, Hall M, . Impact of the Choosing Wisely^®^ Campaign recommendations for hospitalized children on clinical practice: trends from 2008 to 2017. J Hosp Med. 2020;15(2):68-74. doi:10.12788/jhm.3291 31532743

[16] Chua KP, Conti RM, Freed GL. Appropriately framing child health care spending: a prerequisite for value improvement. JAMA. 2018;319(11):1087-1088. doi:10.1001/jama.2018.0014 29482195

[17] Centers for Medicare and Medicaid Services. National health expenditure data: historical. Updated December 16, 2020. Accessed November 9, 2020. https://www.cms.gov/Research-Statistics-Data-and-Systems/Statistics-Trends-and-Reports/NationalHealthExpendData/NationalHealthAccountsHistorical

[18] House SA, Marin JR, Hall M, Ralston SL. Trends over time in use of nonrecommended tests and treatments since publication of the American Academy of Pediatrics bronchiolitis guideline. JAMA Netw Open. 2021;4(2):e2037356. doi:10.1001/jamanetworkopen.2020.37356 33587138 PMC7885040

[19] Parikh K, Hall M, Mittal V, . Establishing benchmarks for the hospitalized care of children with asthma, bronchiolitis, and pneumonia. Pediatrics. 2014;134(3):555-562. doi:10.1542/peds.2014-1052 25136044

[20] Cohen E, Rodean J, Diong C, . Low-value diagnostic imaging use in the pediatric emergency department in the United States and Canada. JAMA Pediatr. 2019;173(8):e191439. doi:10.1001/jamapediatrics.2019.1439 31157877 PMC6547126

[21] Marin JR, Hollander MAG, Ray KN, Donohue JM, Cole ES. Low-value diagnostic imaging in children with Medicaid. J Pediatr. 2021;235:253-263. doi:10.1016/j.jpeds.2021.02.003 33556364 PMC8316256

[22] National Quality Forum. Measure sets and measurement systems: multistakeholder guidance for design and evaluation. July 2020. Accessed August 15, 2020. https://www.qualityforum.org/Publications/2020/07/Measure_Sets_and_Measurement_Systems__Multistakeholder_Guidance_for_Design_and_Evaluation.aspx

[23] House SA, Coon ER, Schroeder AR, Ralston SL. Categorization of national pediatric quality measures. Pediatrics. 2017;139(4):e20163269. doi:10.1542/peds.2016-3269 28298481

[24] Equator Network. The Strengthening the Reporting of Observational Studies in Epidemiology (STROBE) Statement: guidelines for reporting observational studies. Accessed November 11, 2019. https://www.equator-network.org/reporting-guidelines/strobe/

[25] Greiner A. CHA measures selection toolkit. Paper presented at: Children's Hospital Association Quality and Safety in Children's Healthcare Conference; March 9, 2016; New Orleans, Louisiana. Accessed January 15, 2018. https://www.childrenshospitals.org/-/media/Files/CHA/Main/Events/2016/Conferences/Quality-2016/Sessions/Qual16_curatedC5_CHA-Measures-Selection-Toolkit.pdf

[26] American Board of Internal Medicine Foundation. Choosing Wisely: clinician lists. Accessed October 1, 2018. https://www.choosingwisely.org/clinician-lists/

[27] Agency for Healthcare Research and Quality. Pediatric Quality Measures Program: all PQMP measures. Accessed October 1, 2018. https://www.ahrq.gov/pqmp/measures/all-pqmp-measures.html

[28] Mangione-Smith R, Roth CP, Britto MT, . Development and testing of the Pediatric Respiratory Illness Measurement System (PRIMES) quality indicators. Hosp Pediatr. 2017;7(3):125-133. doi:10.1542/hpeds.2016-0182 28223319

[29] Mangione-Smith R, Zhou C, Williams DJ, ; Pediatric Research in Inpatient Settings (PRIS) Network. Pediatric Respiratory Illness Measurement System (PRIMES) scores and outcomes. Pediatrics. 2019;144(2):e20190242. doi:10.1542/peds.2019-0242 31350359 PMC6855826

[30] Feudtner C, Feinstein JA, Zhong W, Hall M, Dai D. Pediatric complex chronic conditions classification system version 2: updated for *ICD-10* and complex medical technology dependence and transplantation. BMC Pediatr. 2014;14:199. doi:10.1186/1471-2431-14-199 25102958 PMC4134331

[31] Children's Hospital Association. High-intensity neurologic impairment codes. Accessed July 11, 2019. https://www.childrenshospitals.org/Research-and-Data/Pediatric-Data-and-Trends/2019/High-Intensity-Neurologic-Impairment-Codes

[32] Keren R, Luan X, Localio R, ; Pediatric Research in Inpatient Settings (PRIS) Network. Prioritization of comparative effectiveness research topics in hospital pediatrics. Arch Pediatr Adolesc Med. 2012;166(12):1155-1164. doi:10.1001/archpediatrics.2012.1266 23027409

[33] Koehlmoos TP, Madsen C, Banaag A, Li Q, Schoenfeld AJ, Weissman JS. Use of low-value pediatric services in the military health system. BMC Health Serv Res. 2020;20(1):770. doi:10.1186/s12913-020-05640-5 32819375 PMC7441552

[34] Gill PJAM, Anwar MR, Thavam T, ; Pediatric Research in Inpatient Setting (PRIS) Network. Identifying conditions with high prevalence, cost, and variation in cost in US children’s hospitals. JAMA Netw Open. 2021;4(7):e2117816. doi:10.1001/jamanetworkopen.2021.17816 34309667 PMC8314139

[35] American Board of Internal Medicine Foundation. Choosing Wisely: Pediatric Hospital Medicine—SHM, AAP, APA: five things physicians and patients should question. January 11, 2021. Accessed September 15, 2021. https://www.choosingwisely.org/societies/pediatric-hospital-medicine-shm-aap-apa/

[36] Neuman MI, Hall M, Lipsett SC, ; Pediatric Research in Inpatient Settings Network. Utility of blood culture among children hospitalized with community-acquired pneumonia. Pediatrics. 2017;140(3):e20171013. doi:10.1542/peds.2017-1013 28835382 PMC5574722

[37] Neuman MI, Shah SS, Shapiro DJ, Hersh AL. Emergency department management of childhood pneumonia in the United States prior to publication of national guidelines. Acad Emerg Med. 2013;20(3):240-246. doi:10.1111/acem.12088 23517255

[38] Milner TL, McCulloh R, Koster M, Biondi E, Hill V, Ralston S. Antibiotic prescribing patterns across the continuum of care for children hospitalized with community-acquired pneumonia. Pediatr Emerg Care. 2018;34(1):e7-e10. doi:10.1097/PEC.0000000000000598 26555311

[39] Florin TA, French B, Zorc JJ, Alpern ER, Shah SS. Variation in emergency department diagnostic testing and disposition outcomes in pneumonia. Pediatrics. 2013;132(2):237-244. doi:10.1542/peds.2013-0179 23878049

[40] Shah SS, Dugan MH, Bell LM, . Blood cultures in the emergency department evaluation of childhood pneumonia. Pediatr Infect Dis J. 2011;30(6):475-479. doi:10.1097/INF.0b013e31820a5adb 21206393 PMC3097278

[41] Rosen R, Vandenplas Y, Singendonk M, . Pediatric gastroesophageal reflux clinical practice guidelines: joint recommendations of the North American Society for Pediatric Gastroenterology, Hepatology, and Nutrition and the European Society for Pediatric Gastroenterology, Hepatology, and Nutrition. J Pediatr Gastroenterol Nutr. 2018;66(3):516-554. doi:10.1097/MPG.0000000000001889 29470322 PMC5958910

[42] Toteja N, Gallego JA, Saito E, . Prevalence and correlates of antipsychotic polypharmacy in children and adolescents receiving antipsychotic treatment. Int J Neuropsychopharmacol. 2014;17(7):1095-1105. doi:10.1017/S1461145712001320 23673334 PMC4010557

[43] Tural Hesapcioglu S, Ceylan MF, Kandemir G, Kasak M, Sen CP, Correll CU. Frequency and correlates of acute dystonic reactions after antipsychotic initiation in 441 children and adolescents. J Child Adolesc Psychopharmacol. 2020;30(6):366-375. doi:10.1089/cap.2019.0123 32255662

[44] UpToDate. Acute rheumatic fever: epidemiology and pathogenesis. Updated October 5, 2020. Accessed September 10, 2021. https://www.uptodate.com/contents/acute-rheumatic-fever-epidemiology-andpathogenesis?search=acute%20rheumatic%20fever&source=search_result&selectedTitle=3~92&usage_type=default&display_rank=3

[45] Ralph AP, Carapetis JR. Group A streptococcal diseases and their global burden. Curr Top Microbiol Immunol. 2013;368:1-27. doi:10.1007/82_2012_280 23242849

[46] Berwick DM, Hackbarth AD. Eliminating waste in US health care. JAMA. 2012;307(14):1513-1516. doi:10.1001/jama.2012.362 22419800

