# Supplementary material for: Development and Use of a Calculator to Measure Pediatric Low-Value Care Delivered in US Children’s Hospitals
Source: JAMA Netw Open. 2021 Dec 30;4(12):e2135184. doi: 10.1001/jamanetworkopen.2021.35184 (PMC8719236; doi:10.1001/jamanetworkopen.2021.35184)
Supplement: Supplement. — eTable. Measure Definitions and Sources [file jamanetwopen-e2135184-s001.pdf]

Supplementary Online Content

House SA, Hall M, Ralston SL, et al. Development and use of a calculator to measure pediatric low-value care delivered in US children's hospitals. *JAMA Netw Open*. 2021;4(12):e2135184. doi:10.1001/jamanetworkopen.2021.35184

eTable. Measure Definitions and Sources

This supplementary material has been provided by the authors to give readers additional information about their work.

© 2021 House SA et al. *JAMA Network Open*.

eTable. Measure Definitions and Sources

For all measures, encounters for patients >18 years and patients with codes for complex chronic conditions or neurologic impairment documented within the year prior to the encounter were excluded. For inpatients, those with an All-Patient Refined Diagnosis Related Group (3M) extreme severity of illness and those admitted to an intensive care unit at any point during hospitalization (with the exception of NICU-specific measures) were also excluded. *International Classification of Diseases, Tenth Revision, Clinical Modification* (ICD-10-CM) used to determine a measure denominator were primary diagnoses only unless otherwise specified. PRIMES = Pediatric Respiratory Illness Measurement System. PQMP = Pediatric Quality Measures Program.

Condition	Measure and Setting	Measure Numerator	Coding Parameters for Inclusion in Measure Denominator	Age Parameters for Inclusion in Measure Denominator	Measure-Specific Denominator Exclusions	Measure Source
Bronchiolitis	Children diagnosed with bronchiolitis should not routinely receive treatment with bronchodilators (ED and Hospitalized)	Encounters with bronchodilator administered	ICD-10-CM codes for bronchiolitis	≥1 month and <24 months	ICD-10-CM codes for asthma, pneumonia, croup	Choosing Wisely, Chua et al.
	Children diagnosed with bronchiolitis should not be treated with antibiotic medications unless they are also diagnosed with a possible bacterial infection (ED and Hospitalized)	Encounters with antibiotic administered	ICD-10-CM codes for bronchiolitis	≥2 months and <24 months	ICD-10-CM codes for asthma, pneumonia, croup, acute otitis media, sinusitis, urinary tract infection, Group A strep pharyngitis, sepsis/bacteremia, skin/soft tissue infections, miscellaneous infections, head/neck infections	Choosing Wisely, PRIMES
	Children diagnosed with bronchiolitis should not be treated with corticosteroids (ED and Hospitalized)	Encounters with inhaled or systemic steroid administered	ICD-10-CM codes for bronchiolitis	≥1 month and <24 months	ICD-10-CM codes for asthma, pneumonia, croup	Choosing Wisely, PRIMES, Chua et al.
	Children diagnosed with bronchiolitis should not have a	Encounters with chest x-ray performed	ICD-10-CM codes for bronchiolitis	≥1 month and <24 months	ICD-10-CM codes for asthma, pneumonia, croup	Choosing Wisely, PRIMES

© 2021 House SA et al. *JAMA Network Open*.

	chest x-ray performed. (ED and Hospitalized)					
	Children diagnosed with bronchiolitis should not have bacterial blood cultures performed. (ED and Hospitalized)	Encounters with blood cultures performed	ICD-10-CM codes for bronchiolitis	>2 months and <24 months	ICD-10-CM codes for asthma, pneumonia, croup	PRIMES
Asthma	Children admitted to the hospital with acute exacerbation of asthma should not receive ipratropium bromide after 24 hours of hospitalization. (Hospitalized)	Encounters with ipratropium bromide administered after 24 hours of admission	ICD-10-CM codes for asthma	>24 months to <18 years	ICD-10-CM codes for bronchiolitis, pneumonia, croup	PRIMES
	Children diagnosed with asthma should not be treated with antibiotic medications unless they are also diagnosed with a possible bacterial infection. (ED and Hospitalized)	Encounters with antibiotics administered	ICD-10-CM codes for asthma	>24 months to <18 years	ICD-10-CM codes for bronchiolitis, pneumonia, croup, acute otitis media, sinusitis, urinary tract infection, Group A strep pharyngitis, sepsis/bacteremia, skin/soft tissue infections, miscellaneous infections, head/neck infections	PRIMES
	Children diagnosed with asthma should not routinely have a chest x-ray performed. (ED and Hospitalized)	Encounters with chest x-ray performed	ICD-10-CM codes for asthma	>24 months to <18 years	ICD-10-CM codes for bronchiolitis, pneumonia, croup	Choosing Wisely
Pneumonia	Children diagnosed with uncomplicated community acquired pneumonia (CAP) should not have C-reactive protein (CRP)	Encounters with either ESR or CRP performed	ICD-10-CM codes for pneumonia	≥3 months to <18 years	ICD-10-CM codes for bronchiolitis, asthma, croup, sepsis/Bacteremia. Complicated pneumonia as defined	PRIMES

© 2021 House SA et al. *JAMA Network Open*.

	and erythrocyte sedimentation rate (ESR) tests performed. (ED and Hospitalized)				by diagnostic coding strategy for complicated pneumonia measure	
	Children diagnosed with uncomplicated community acquired pneumonia (CAP) should not be treated with antibiotic therapies broader than ampicillin. (ED and Hospitalized)	Encounters with antibiotics other than amoxicillin or ampicillin administered	ICD-10-CM codes for pneumonia	≥3 months to <18 years	ICD-10-CM codes for bronchiolitis, asthma, croup, underimmunization, sepsis/bacteremia, Complicated pneumonia as defined by diagnostic coding strategy for complicated pneumonia measure	Choosing Wisely, House et al.
	Children diagnosed with uncomplicated community acquired pneumonia (CAP) should not routinely have bacterial blood cultures performed. (ED and Hospitalized)	Encounters with blood cultures performed	ICD-10-CM codes for pneumonia	≥3 months to <18 years	ICD-10-CM codes for bronchiolitis, asthma, croup. Complicated pneumonia as defined by diagnostic coding strategy for complicated pneumonia measure	PRIMES
	Children diagnosed with complicated pneumonia should not have peripherally inserted central lines (PICC) or central venous lines (CVL) placed for extended intravenous antibiotic therapy; oral conversion to antibiotics is preferred to PICC/CVL usage in children. (Hospitalized)	Encounters with PICC/ CVL lines placed	ICD-10-CM codes for complicated pneumonia or ICD-10-CM codes for pneumonia with a procedural code indicating pleural drainage or chest tube placement	<18 years	ICD-10-CM codes for bronchiolitis, asthma, croup	Choosing Wisely

© 2021 House SA et al. *JAMA Network Open*.

Viral Respiratory Infection	Children diagnosed with viral respiratory infections should not be treated with antibiotic medications unless they are also diagnosed with a possible bacterial infection.<br>(ED and Hospitalized)	Encounters with antibiotics administered	ICD-10-CM codes for for upper respiratory infection, other lower respiratory tract infections, bronchiolitis, cough, congestion, viral pneumonia	≥2 months to <18 years	ICD-10-CM codes for bronchiolitis, pneumonia, croup, acute otitis media, sinusitis, urinary tract infection, Group A strep pharyngitis, sepsis/bacteremia, skin/soft tissue infections, miscellaneous infections, head/neck infections	Choosing Wisely, Chua et al., House et al.
Pharyngitis	Children <3 yrs. seen and treated in the ED for pharyngitis should not routinely receive testing for Group A Strep (GAS) pharyngitis unless other risk factors are present.<br>(ED)	Encounters rapid streptococcal testing performed	ICD-10-CM codes for pharyngitis	<3 years	None	Chua et al.
Gastroesophageal reflux	Infants <1 year should not be routinely be treated with acid suppression therapy.<br>(ED and Hospitalized)	Encounters with acid suppression therapy administered	ICD-10-CM codes for esophageal reflux in primary or secondary diagnostic position	<1 year	ICD-10-CM codes for esophagitis, peptic ulcer disease, gastritis and/or duodenitis	Choosing Wisely, Chua et al.
Head Injury	Children seen and treated in the ED for minor head injuries should not routinely receive a head/brain computed tomography (CT) scan.<br>(ED)	Encounters with head CT performed	ICD-10-CM codes for head injury or concussion without loss of consciousness	≥2 years to <18 years	ICD-10-CM codes for skull fracture, concussion with loss of consciousness, traumatic avulsion/amputation, crush injury	Choosing Wisely

© 2021 House SA et al. *JAMA Network Open*.

Headache	Children seen and treated in the ED for acute atraumatic primary headache should not routinely receive MRI. (ED)	Encounters with MRI performed	ICD-10-CM codes for headache, migraine, headache syndrome, complicated headache syndromes	≥4 years to <18 years	ICD-10-CM codes for post-traumatic headache, thunderclap headache, concussion, skull fracture	PQMP, Chua et al.
	Children seen and treated in the ED for acute atraumatic primary headache should not routinely receive CT. (ED)	Encounters with CT performed	ICD-10-CM codes for headache, migraine, headache syndrome, complicated headache syndromes	≥4 years to <18 years	ICD-10-CM codes for post-traumatic headache, thunderclap headache, concussion, skull fracture	PQMP, Chua et al., House et al.
Febrile Seizure	Children diagnosed with a simple febrile seizure should not routinely receive computed tomography (CT) imaging of the head. (ED and Hospitalized)	Encounters with MRI performed	ICD-10-CM code for simple febrile seizure	≥6 months to <4 years	ICD-10-CM codes for meningitis, encephalitis, complex febrile seizure, post-traumatic seizure or procedural code for lumbar puncture	Choosing Wisely, PQMP, Chua et al.
	Children diagnosed with a simple febrile seizure should not routinely receive magnetic resonance imaging (MRI)of the head. (ED and Hospitalized)	Encounters with CT performed	ICD-10-CM code for simple febrile seizure	≥6 months to <4 years	ICD-10-CM codes for meningitis, encephalitis, complex febrile seizure, post-traumatic seizure or procedural code for lumbar puncture	Choosing Wisely, PQMP, Chua et al.
	Children diagnosed with a simple febrile seizure should not routinely have electrolyte testing	Encounters with electrolyte testing performed	ICD-10-CM code for simple febrile seizure	≥6 months to <4 years	ICD-10-CM codes for meningitis, encephalitis, complex febrile seizure, post-traumatic seizure,	Chua et al.

© 2021 House SA et al. *JAMA Network Open*.

	performed for the sole purpose of identifying the cause of the seizure. (ED and Hospitalized)				vomiting, diarrhea, or dehydration or procedural code for lumbar puncture	
	Children diagnosed with a simple febrile seizure should not routinely have complete blood count (CBC) testing performed for the sole purpose of identifying the cause of the seizure. (ED and Hospitalized)	Encounters with complete blood cell count performed	ICD-10-CM code for simple febrile seizure	≥6 months to <4 years	ICD-10-CM codes for meningitis, encephalitis, complex febrile seizure, post-traumatic seizure, vomiting, diarrhea, or dehydration or procedural code for lumbar puncture	Chua et al.
Seizure	Children diagnosed with their first generalized afebrile atraumatic seizure should not have computed tomography (CT) imaging of the head performed. (ED and Hospitalized)	Encounters with head CT performed	ICD-10-CM codes for seizures *without* ICD-10-CM code for seizure documented for up to one year prior to the included encounter	≥1 years to <18 years	ICD-10-CM codes for meningitis, encephalitis, complex febrile seizure, post-traumatic seizure, partial seizure, fever, suspected child abuse or neglect, skull/facial fracture, concussion, brain abscess, status epilepticus, epilepsy or procedural code for lumbar puncture	PQMP
Abdominal pain	Children with abdominal pain should not routinely have computed tomography (CT) imaging of the abdomen performed unless other indications are present. (ED and Hospitalized)	Encounters with abdominal CT performed	ICD-10-CM codes for abdominal pain, constipation	≥3 to <18 years	ICD-10-CM codes for bowel obstruction, abdominal/spinal/pelvic trauma, pregnancy	Choosing Wisely

© 2021 House SA et al. *JAMA Network Open*.

Bone and Joint infections	Children diagnosed with bone and joint infections should not have peripherally inserted central lines (PICC) or central venous lines (CVL) placed for extended intravenous antibiotic therapy; oral conversion to antibiotics is preferred to PICC/CVL usage in children. (Bone and Joint infections measure) (Hospitalized)	Encounters with central line placement	ICD-10-CM codes for osteomyelitis and septic arthritis	<18 years	None	Choosing Wisely
Ruptured appendicitis	Children diagnosed with ruptured appendicitis should not have peripherally inserted central lines (PICC) or central venous lines (CVL) placed for extended intravenous antibiotic therapy; oral conversion to antibiotics is preferred to PICC/CVL usage in children. (Ruptured appendicitis infections measure) (Hospitalized)	Encounters with central line placement	ICD-10-CM codes for ruptured appendicitis	<18 years	None	Choosing Wisely
Behavioral Health	Children receiving antipsychotic medications should not routinely receive two or more antipsychotic medications	Encounters with 2 or more antipsychotic medications administered	Clinical transaction classification (CTC) code for any antipsychotic medication	≥1 years to <18 years	None	PQMP, House et al.

© 2021 House SA et al. *JAMA Network Open*.

	concurrently. (Hospitalized)					
	Children <5 years should not routinely receive antipsychotic medications. (Hospitalized)	Encounters with any antipsychotic medication administered	All eligible encounters within age parameters	≥1 years to <5 years	None	PQMP, House et al.
NICU Infection General	Infants in the NICU should not receive vancomycin or carbapenems unless there is a known risk for resistant pathogens. (Hospitalized – NICU)	Encounters with vancomycin or carbapenem agents administered	All eligible encounters for infants admitted to the NICU	<1 year	ICD-10-CM codes for methicillin resistant staphylococcal aureus, extended spectrum beta-lactamase resistance, multi-drug resistance	Choosing Wisely
NICU GERD	Infants in the NICU should not receive antireflux medications for treatment of symptomatic GERD or for the treatment of apnea and desaturation. (Hospitalized – NICU)	Encounters with acid suppression administered	ICD-10-CM Codes for gastroesophageal reflux and apnea of prematurity for infants admitted to the NICU	<1 year	ICD-10-CM codes for apparent life-threatening event, esophagitis, peptic ulcer disease, gastritis and/or duodenitis	Choosing Wisely

© 2021 House SA et al. *JAMA Network Open*.
